# Accounting carbon emission and proposals for their reduction at a university campus in China

**DOI:** 10.1038/s41598-025-23719-z

**Published:** 2026-03-23

**Authors:** Jianfei Liu, Xinyu Mao, Huihui Wang

**Affiliations:** School of civil engineer, Henan Polytechnic University, Jiaozuo, 454000 China

**Keywords:** Universities, Carbon emission calculation, Carbon transfer, Emission reduction measures, Energy and society, Sustainability

## Abstract

In the context of global sustainable development, various industries actively account for their carbon emission to address climate change effectively. University campuses should also actively participate in this pursuit. The carbon emission calculation method introduced by the Greenhouse Gas Protocol was applied in the paper, with Henan Polytechnic University’s south campus being used as a case study. Energy and food consumption data were collected from the school’s relevant offices, and other data were obtained through questionnaires and multiplied by the corresponding carbon emission factors to calculate the carbon emission of the campus in 2019. Results showed that the carbon emission were 65327.44 tons, with a per capita of 1.51 tons. The analysis revealed that electricity and heating systems constituted the primary contributors to carbon emission. Heating emission profiles exhibited pronounced seasonal dependency, while electricity carbon emission ‌varied‌ across different functional areas. Two innovative findings were established: carbon transfer from transportation to the building sector was driven by accelerated electric vehicle adoption, and a context-specific energy coupling system was proposed, with emission reductions being quantified through localized analysis. The context-specific short-term and long-term carbon reduction solutions are proposed. The carbon emission accounting conducted and targeted carbon reduction measures proposed in this study are considered to provide not only a reference path for universities to achieve carbon neutrality but also a theoretical foundation and methodological guidance for carbon emission reduction.

**Supplementary Information:**

The online version contains supplementary material available at 10.1038/s41598-025-23719-z.

## Introduction

Greenhouse gas (GHG) is a broad concept, which includes all the gases that can cause the earth’s surface temperature to rise. CO_2_ is the most common GHG with the highest proportion. The escalating climate warming and frequent occurrence of catastrophic global climate phenomena had rendered GHG emission reduction and low-carbon society construction a globally recognized priority^1^. Sustainable development encompassed all facets of socioeconomic systems, spanning hierarchical units from national/regional levels to cross-sectoral entities including governmental bodies, industrial plants, commercial enterprises, and educational institutions. Within this framework, universities historically fulfilled unique roles in sustainability advancement. Through campus-based sustainability initiatives, students were being imperceptibly educated to develop environmental stewardship while simultaneously functioning as societal transmitters of sustainable development principles through their academic and professional trajectories^2^. Consequently, institutional sustainability practices held not only significant practical value for resource-efficient societal development but also demonstrated profound pedagogical implications.

The sustainable development of universities has gradually become a hot issue of global concern. Modern university campuses are increasingly emerging in the form of large-scale university towns with large populations and a higher personnel density than cities. As the most active hubs for scientific research and social activities, these campuses—characterized by extended lifecycles, organizational concentration, and high population density—serve as ideal settings for researching carbon emission strategies, energy-saving behaviors, and related studies. They should pioneer efforts in both research and practical implementation of CO₂ reduction, acting as concrete manifestations of carbon emissions at specific spatial scales. Through their own carbon reduction practices, universities propagate green, low-carbon, energy-saving, and eco-friendly principles, transforming into environmental platforms that disseminate low-carbon ethos. Recently, 1,050 universities from 68 countries committed to becoming green campuses by 2050 at the Times Higher Education Climate Impact Forum^3^. Most universities’ carbon emission reduction plans focus on the construction of low-carbon campuses, lacking some data support. Knowing one’s carbon emission is the first step for formulating energy conservation and emission reduction measures. Thus, the calculation of campus carbon emission has become an urgent task. For universities, different scholars use different energy sources and select different carbon emission factors when calculating campus statistics. As a result, the comparison of calculation results was ‌moderately impeded‌, and limited literature was systematically derived from the analysis of differentiated carbon emission sources. Campus carbon emission research was concentrated on total energy consumption and building operations analysis, while cross-departmental emission characteristic comparisons remained underexplored. Campuses were as undifferentiated entities in prior frameworks, leading to obscured carbon transfer phenomena between departments. Current research attempted to clarify the status quo of carbon emissions in universities and explored campus carbon reduction measures from diverse perspectives, yet a consensus on accounting boundaries and theoretical frameworks for campus carbon emissions remained elusive. This gap consequently hindered comprehensive considerations of various policy initiatives during university carbon reduction planning. This paper describes how to establish a replicable assessment model based on the GHG protocol, which integrates Scope 3 emissions (including student commuting, rarely addressed in previous campus carbon emission studies). Moreover, while implementable emission reduction measures were generally absent from existing literature or remained conceptual in nature, a context-specific campus energy solution was systematically formulated through multi-energy system optimization.

This study takes Henan Polytechnic University (HPU) as an example to achieve the following objectives: (1) calculating the accounting schemes for carbon emission in three areas based on the GHG Protocol, (2) identifying key emission sources and analyzing their emission characteristics, and (3) proposing specific measures for campus carbon reduction tailored to local conditions.

Two methodological innovations were advanced in campus carbon management research through this study. One innovation was the development of a refined classification system that revealed an emerging carbon transfer phenomenon - where transportation field emissions were observed to progressively migrate to building field due to accelerated electric vehicle adoption. The other innovation was the establishment of a context-specific multi-energy complementary optimization framework through energy flow analysis, diverging from conventional studies constrained to emission accounting. The results of this study can provide a reference for universities to compile carbon emission inventories and conduct carbon emission accounting. This study identifies methodological constraints in carbon accounting stemming from institutional complexities in system boundaries‌ and inadequate dynamic coupling of artificial intelligence driven multi-scenario decarbonization optimization mechanisms across scales‌, which will be the future research direction. In addressing global warming and researching the establishment of a low-carbon development and carbon neutrality pathway framework for universities, this study proposes that achieving campus carbon neutrality can not only provide reference for low-carbon green campus construction and management, but also offer scientific reference for reducing carbon emissions and achieving carbon neutrality across regions and industries.

## Literature review

The GHG Protocol jointly developed by the World Resources Institute and the World Business Council for Sustainable Development, is an international standard for greenhouse gas accounting and reporting, which is widely used in carbon emission management of enterprises, governments and various organizations. GHG Protocol divides carbon emission into Scope 1, Scope 2 and Scope 3^4^. Most early studies limited the boundary to direct emissions (Scope 1) and indirect energy related emissions (Scope 2) within the physical boundaries of the study area, but in recent years, studies have gradually included more complex emissions (Scope 3)^5^. For physical boundaries, most studies currently limit the boundary to the “red line” of the campus, and do not count the carbon emission generated by teachers and students outside the campus. For the time boundary, as a small composite ecosystem, the main carbon emission measurement cycle of universities is generally recognized to include the construction period and the operation period. The construction period includes the energy consumption in the construction phase, as well as the embodied carbon of building materials. The carbon emission during the operation period includes the energy consumption (electricity, water, gas, oil, etc.) in the campus.

The carbon emission calculation methods usually include Intergovernmental Panel on Climate Change (IPCC) inventory method, input-output method and life cycle assessment (LCA) method. The IPCC is positioned at the national/regional level for the preparation of greenhouse gas inventories, which are usually used for international implementation and policy formulation^6^. The calculation scope covers the total emission statistics of national energy, industry, agriculture and other sectors. Emission factor approach is a carbon emission estimation method proposed by the IPCC and is widely used at present. The basic idea is to construct activity data and emission factor for each emission source according to the carbon emission inventory list. The emission factor approach can reflect the actual emissions of carbon emission sites. The method can distinguish the differences between various facilities and those between individual and partial equipment.

The input-output method is a quantitative analysis method used to evaluate the input-output relationship between different sectors and elements in the social economy^7^. In the context of input-output analysis, the Leontief inverse matrix ‌was employed as‌ the core mathematical instrument to fundamentally ‌reveal‌ the transmission pathways of lifecycle carbon emissions driven by final demand^8^. Since the method is directly based on the input-output table published by the state, it is more suitable for carbon emission research at the macro level. This method supports the evaluation of the pulling effect of industrial structure on carbon emission at the national or regional level, such as analyzing the carbon spillover impact of high carbon industries on other industries, and providing data support for the development of carbon peak paths and optimization of energy policies. Through the interregional input-output model, the carbon emission transfer path can be identified, providing a basis for regional collaborative emission reduction and carbon responsibility division. This method, combined with the economic forecasting model, can simulate the impact of different policies on the overall carbon emission and assess the emission reduction potential of the policy portfolio^9^.

LCA method is positioned at the environmental impact assessment of the whole life cycle of products or services, including the carbon emission of the whole process from the acquisition, production, use to waste of raw materials, which is usually used for product carbon footprint certification, green design optimization and environmental label development^10^. ‌Environmental design of industrial products methodology ‌^11^ or ‌hybrid life cycle assessment ‌^12^ was established analytical frameworks for comprehensive emission quantification. The literature^13^ summarized 135 applications of this method and showed that in the past 20 years, the application research of LCA in campus carbon emission calculation gradually increased, and the research field was divided into eight fields. The literature^14^ pointed out 826 building carbon emission calculation cases from 161 global studies, analyzed the calculation concept, calculation method and basic parameters, and proposed corresponding emission reduction strategies. It can be seen that LCA, as a basic method, can be used for systematic optimization of emission reduction strategies only after detailed analysis of specific situations based on the whole life cycle of buildings.

Achieving carbon neutrality goals necessitates the acquisition of comprehensive data on campus carbon emission sources and the systematic analysis of the proportional contributions from distinct emission categories. Papers on the calculation of campus carbon emission have been summarized in some review papers^15–17^. However, few universities offer their carbon emission data even though the number of universities worldwide currently exceeds 45,000. Authors use “‘college’ OR ‘campus’ OR ‘university’ OR ‘higher education institutions’” AND “‘carbon emission’ OR ‘carbon footprint’ OR ‘greenhouse gas’” as the keyword to search in the Web of Science. After screening irrelevant literature, 59 papers of accounting data on campus carbon emission have been obtained since 2010. This observation indicates that scholars pay considerable attention to their research fields and little attention to the carbon emission of their environment. In addition, previous studies primarily focused on carbon emission calculations with scopes 1, 2, and 3 distinctions. This research extended the analysis by systematically classifying and comparing emission sources across these scopes. Regarding low-carbon and sustainable campus construction, some schools in certain countries have developed their unique low-carbon campus construction plans. Some carbon neutrality roadmaps and plans have been successively formulated. Table 1 lists the carbon neutrality action plans of some universities.

**Table 1:** Carbon neutrality action plans of some universities.

University name	Action plan name, year	Goal	Main measures	Ref
Cornell University	Climate Action Plan,2009	The goal of reducing carbon-based emissions from the Ithaca campus to net zero by the year 2050	Cornell has embraced a sustainability framework that incorporates the three key areas of environment, economy, and equity, and adds a fourth area of consideration which helps us examine how we can best contribute to a more sustainable world as an educational institution.	^18^
University of California, Berkeley	UC Berkeley Sustainability Plan, 2009	Climate neutrality from scope 1 and 2 sources by 2025 Climate neutrality from specific scope 3sources by 2050 or sooner	The Plan describes the broad campus commitment to sustainability in five cores are as: Climate & Resiliency, Built & Natural Environment, Sustainable Services, Health &Sustainability, and Culture & Learning. The Plan will guide future work on campus and establish a structure to identify and achieve continuous improvement.	^19^
Harvard University	Sustainability Action Plan,2014	Harvard set a goal to be fossil fuel-free by 2050. As a bridge to reach Goal Zero, Harvard has a short-term objective to be fossil fuel-neutral by 2026. This means Harvard will zero out campus emissions (Scope 1 and Scope 2).	Harvard is addressing climate change and the environment, equity and health in an integrated, interconnected way, rather than as separate issues.	^20^
Stanford University	Energy and Climate Plan,2015	At least net-zero greenhouse gas emissions by 2050	The plan demonstrates long-term cost effectiveness and sustainable natural resource use; guides development of critical campus infrastructure; and reduces economic and regulatory risks to Stanford’s long-term energy supply. It provides a vision for the campus’ energy future while maintaining flexibility through a comprehensive, long-term approach to the challenge of reducing campus emissions.	^21^
The University of Sheffield UK	University Sustainability Strategy, 2019	Being a net-zero carbon University by 2038, and net-zero for Scope 1 and 2 emissions by 2030.	Using research expertise to tackle twenty-first century sustainability challenges Embedding Education for Sustainable Development into all taught courses 100% renewable procured electricity on campus Tough action on high-carbon travel	^22^
London School of Economics and Political Science	Sustainability Strategic Plan,2020	Becoming net zero carbon by 2030 for direct energy use (scope 1 and 2) and by 2050 for all indirect emissions (scope 3)	Adopt a systematic approach to carbon management, based on a hierarchy of options and prioritising reductions. Commission any new construction projects for our campus & residences to be net-zero carbon and meet the highest energy efficiency standards. Invest in further measures to bring our estate to highest energy efficiency standards. Continue to source 100% of the electricity we buy from renewable sources (e.g. solar and wind).	^23^
Massachusetts Institute of Technology	MIT’s Climate Action Plan for the Decade, 2021	Achieve net-zero carbon emission by 2026, with a goal of eliminating direct emissions by 2050	The majority of new reductions will be achieved through new large-scale off-campus renewable energy, storage, and infrastructure projects to neutralize or sequester remaining direct campus greenhouse gas emissions. By creating momentum for large renewable energy projects, MIT can offset its carbon emission while promoting equity, benefiting local economies, and improving public health.	^24^
Delft University of Technology	Climate Action Programme,2021	TU Delft aims to be carbon neutral, climate-adaptive and circular, with contribution to the quality of life and biodiversity, by 2030.	Sustainable procurement: as soon as possible Energy: geothermal energy, renovations and super-sustainable new builds Food & beverage: continue to improve the sustainability of food Mobility: make the campus fossil free, and travel sustainably Data Management: reduce processing energy and use AI to improve campus operations Compensation: green the campus and compensate CO_2_ annually	^25^
University of Brighton	Net-Zero Strategy, 2021	The university has committed to achieve net-zero carbon emission by 2050.	Demand reduction – Improvements to building fabric, energy efficiency and resource optimization to minimize the university’s energy demand. Energy generation – Producing our own clean, affordable energy. Responsible energy procurement – Source our remaining energy needs from sustainable sources.	^26^
The University of Tokyo	UTokyo Climate Action,2022	It is essential to set mid-term targets (50% reduction of the university’s effective CO2 emissions in scopes 1 and 2 by 2030 compared to the fiscal 2013) for 2030, accelerate energy-saving measures and introduce energy-creating equipment to achieve carbon neutrality in 2050	Adopt on-site/offsite energy creation and energy storage Procure renewable energy Emission reduction measures along with measurement methods are being considered for Scope 3 categories.	^27^

In addition to the above action plans announced by colleges and universities, some literatures also summarized the existing carbon neutral plans of universities. Literature^28^ used an organized search strategy for reviewing the most impactful previous studies regarding decarbonizations strategies in campus in different climate contexts and presented a comprehensive overview of influential parameters. Literature^29^ advocated for comprehensive sustainability plans for educational leaders integrating zero-carbon objectives, resilience measures, and active community engagement. Literature^30^ shown that it is necessary to develop a framework that combines the concepts of smart campus and smart energy system to achieve zero emissions on university campuses by studying the energy system on British campuses. Cui^31^ analyzed the literature of the past decade and proposed implementing carbon emission reduction measures in four aspects: technology application, carbon sink function, planning layout, and organizational behavior. Wang^32^ analyzed the carbon emission data of eight universities in China, built a carbon neutral planning framework for universities in China University of Geosciences (Wuhan) with eight contents, and promised to achieve carbon neutrality on campus by 2052. Cai^33^ calculated the campus carbon emissions of Beijing Normal University and proposed two carbon reduction scenarios. These technology roadmaps focus mainly on concepts, ideas, and frameworks and propose some flexible response strategies. The applications of carbon reduction technologies are few. Current research predominantly emphasizes the development of macro-strategic frameworks, with limited empirical investigation into data-driven emission reduction measures. This study employs HPU as a case study to systematically analyze campus carbon emission patterns, proposing differentiated short-term and long-term mitigation solutions. The findings aim to provide actionable insights for advancing low-carbon development in higher education institutions.

## Research methodology

### Research area

HPU is a public university located in Jiaozuo, Henan Province of China. Jiaozuo has a temperate monsoon climate with sufficient sunshine. The annual average temperature is 12.8 ℃‒14.8 ℃. July is the hottest month, and January is the coldest month. The annual atmospheric precipitation is 500–700 mm. HPU has three campuses, and the biggest campus, namely, the south campus, which was built from 2001, is selected as the research campus. The HPU south campus covers an area of 167.52 ha, with a high greening rate, many lawns, and two self-built lakes. In 2019, the south campus had 3113 teachers and staff, 39,747 domestic students, and 277 international students from 30 countries. The campus has 23 teaching schools and 82 undergraduate majors. It includes not only classrooms, dormitories, canteens, laboratories, office buildings, and sports facilities but also extensive vegetation, such as green spaces and trees. Thus, it is a typical example of most campuses in China.

### Research framework

**Fig. 1:**
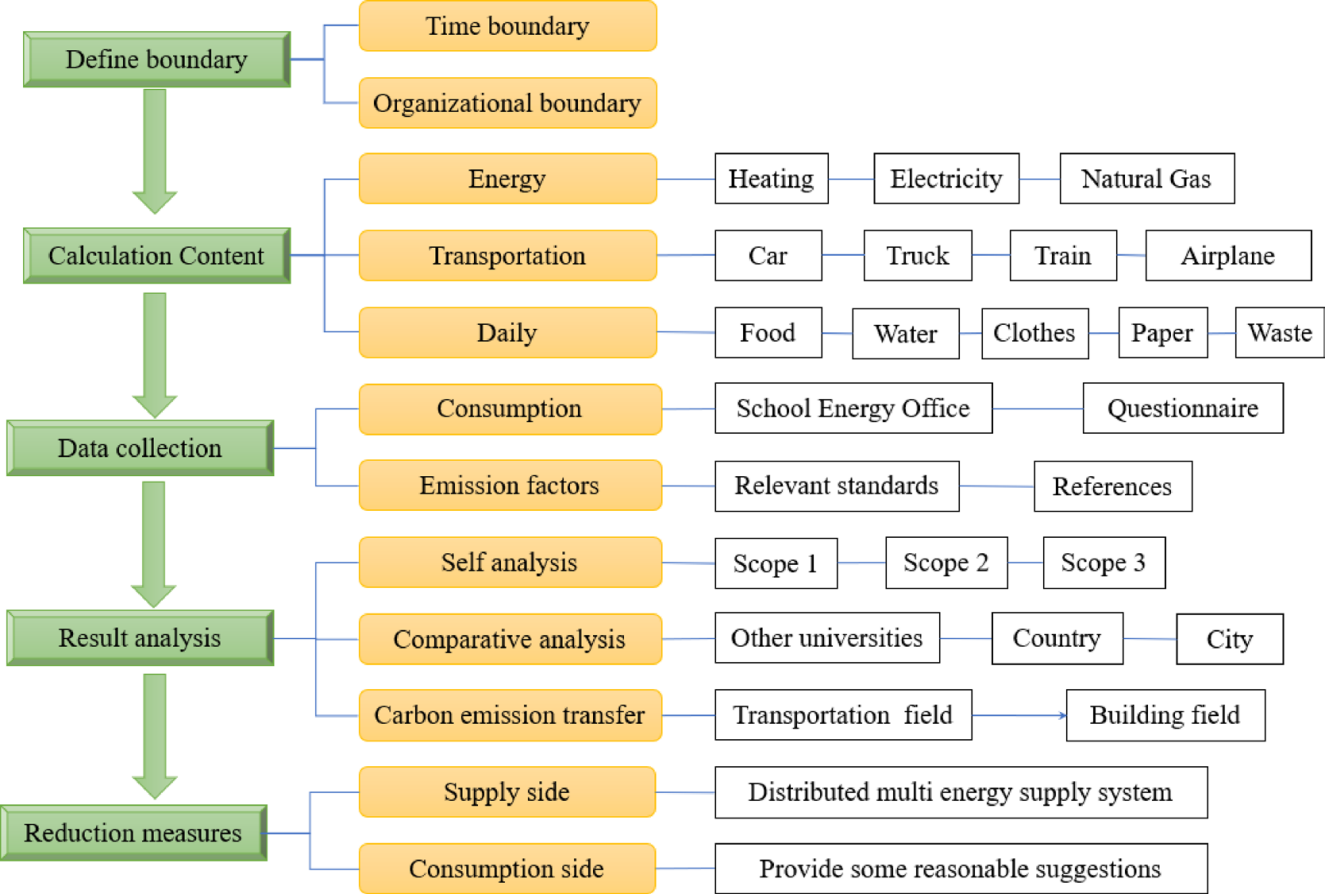
Research framework.

The Research framework details are illustrated in Fig. 1. The study implemented a systematic carbon accounting methodology comprising five sequential phases.

First, spatial and temporal boundaries were rigorously delineated to establish the geographic scope and research period. The carbon emission accounting boundary includes organizational and time boundaries. The organizational boundary is defined as all the buildings and facilities belonging to the school within the space of the campus. Furthermore, the accounting scope of this study is not limited to campus walls but includes the off-campus trips of teachers and students. On the time boundary, the life cycle of the campus includes three stages: construction, operation, and demolition. The year 2019 was selected as the study period—this year offers a complete record of teaching operations and energy consumption characteristics unaffected by pandemic disruptions.

Secondly, a multi-dimensional accounting framework was developed to establish system-wide carbon source inventories spanning building energy end-uses (heating, electricity, natural gas), transportation sectors (including private rides, long-distance passenger transportation, railways, and aviation), and lifestyle consumption domains (including food, water, paper, textiles, and solid waste).

Thirdly, the consumption of energy was obtained from the campus energy office. The data on the food raw material consumption and waste generation of the campus obtained from the school’s catering service center and waste transfer center, respectively. All the above data can be found in Supplementary Material Table S1. Transportation data were surveyed using a questionnaire method to investigate the students’ daily commuting and returning activities and the teachers’ daily commuting and business travel activities. The results of the questionnaire survey on transportation can be found in Table S2 and S3 in the Supplementary Material. The data on transportation modes, distances, and durations were obtained, and the per capita energy consumption of transportation was calculated. The frequency, shipping distance, and parcel weight of student online purchases ‌were examined‌ through a questionnaire survey to quantify this behavior. Detailed results are presented in Table S4 - S6 of the Supplementary Material. The data on office paper consumption were also collected using a survey questionnaire method to investigate the paper consumption of campus students and teachers. Additional survey content can be found in the literature^34^.

Fourthly, carbon emissions were structurally decomposed according to Scopes 1–3 of the GHG Protocol Corporate Standard, with cross-sectional benchmarking conducted against peer institutions, national and city level. In this study, Scope 1 covers the direct emissions from gas consumption and automobiles with fuel inside the boundary. Scope 2 covers the indirect emissions from the campus’s purchased electricity consumption and hot water for heating. Scope 3 covers student commuting, staff commuting, and transport of goods, food, paper, and garbage treatment.

Lastly, spatially heterogeneous characteristics informed the formulation of co-optimized abatement pathways integrating supply-side interventions with demand-side management strategies.

### Carbon emission calculate

The Carbon emission factor method, as an internationally recognized core approach for carbon accounting, traces its methodological origins to the National Greenhouse Gas Inventory Guidelines published by the IPCC. It was subsequently systematized and developed into a standardized accounting framework through the GHG Protocol.

The carbon emission can be calculated for each source using the following formula^4^:

GHG emissions = activity data × emissions factor.

Activity data encompasses critical parameters such as energy consumption (fuel usage, purchased electricity, etc.), production processes (raw material input, etc.), and waste treatment volumes. The selection of emission factor follows a strict hierarchy: priority is given to site-specific emission factor, followed by national/regional emission factors, with international default values from the IPCC emission factor database serving as the last resort. All activity data and emission factor data in this study can be found in supplement material.

## Campus carbon emission accounting

### Analysis of carbon emission

**Fig. 2:**
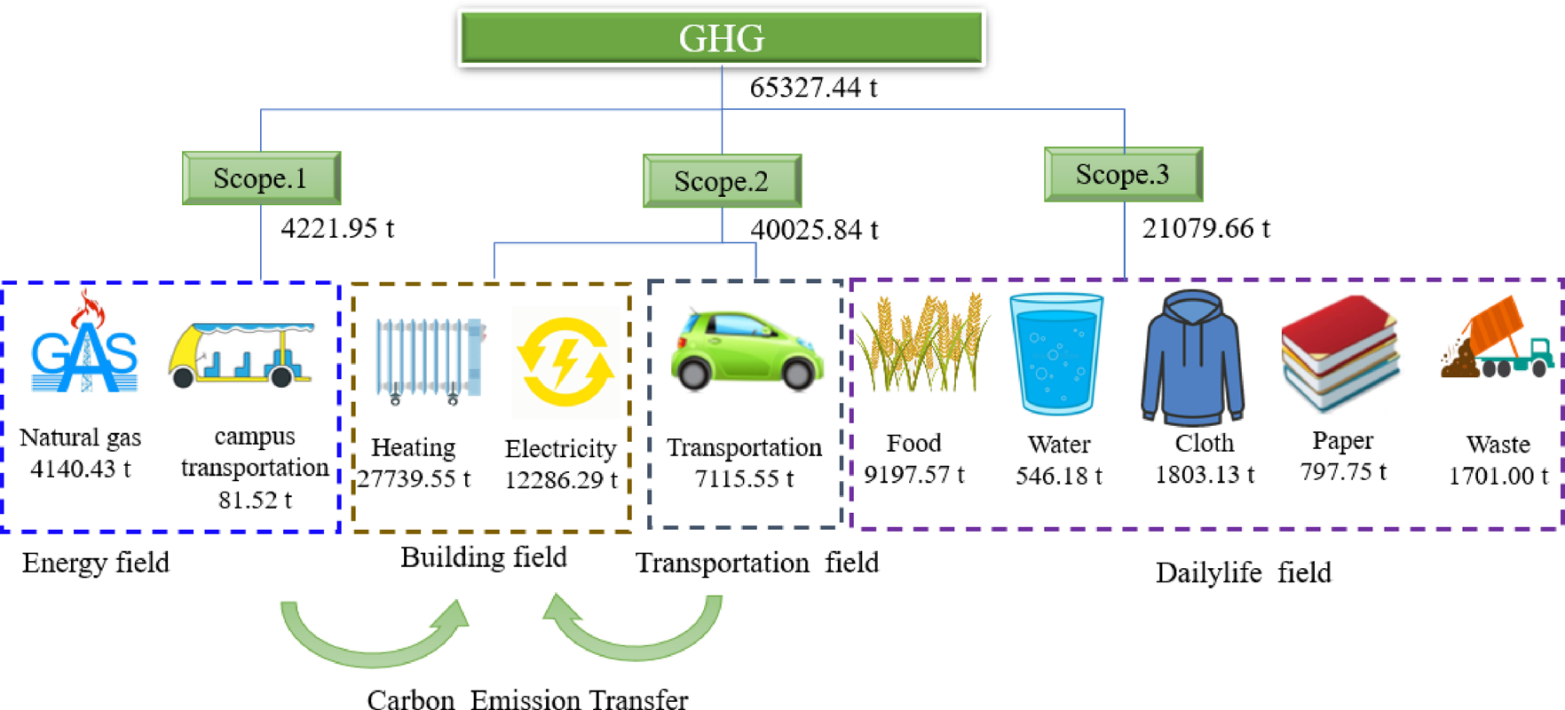
Composition of carbon emission.

The total carbon emission of HPU south campus in 2019 were 65327.44 CO_2_, with a per capita of 1.51 tCO_2_/person. As shown in Fig. 2, the proportions of Scopes 1, 2, and 3 were 6.46%, 61.27%, and 32.27%, respectively.

The total carbon emission of Scope 1 are 4221.95 tons of CO_2_, mainly composed of natural gas consumed by the school canteen and faculty apartments, with a carbon emission of 4140.43 tons of CO_2_. The carbon emission from campus transportation are 81.52 tons of CO_2_, which accounts for a relatively low proportion. The main reason is that the teaching and office areas on campus are relatively concentrated, and the faculty residences are close to the teaching and office areas. Most faculty members use electric vehicles or bicycles, or they walk to school.

Scope 2 covers the indirect emissions of heat used in heating systems and the consumption of electricity purchased by organizations, totaling 40025.84 tCO_2_ emissions. The school adopts the municipal heating network connection method for heating, and the campus has two centralized heat exchange stations. Heating carbon emission has seasonal characteristics, only occurring during the heating season (from November 15th to March 15th of the next year). Heating carbon emission are the primary source of carbon emission for schools, with a total emission of 27739.55 tCO_2_, accounting for 41.13% of the total campus emissions.

**Fig. 3:**
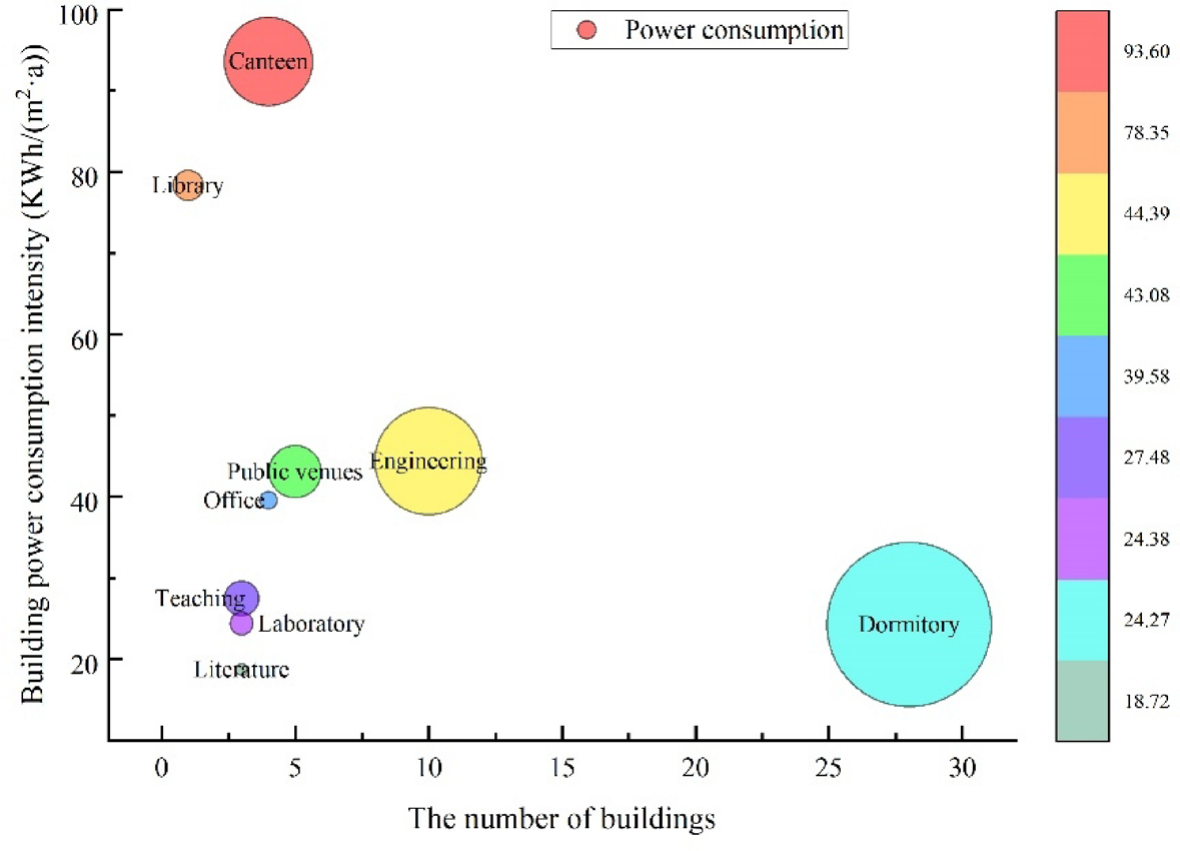
The power consumption intensity with different building.

Electricity, the main type of energy consumption on the campus, is purchased from the social power grid. After voltage reduction in the distribution room, the electricity is distributed to various electricity-consuming units for use, with a total emission of 12286.29 tCO_2_, accounting for 18.22%. The main electrical buildings include teaching buildings, laboratory buildings, restaurants, residential buildings, dormitory buildings, and libraries. The types of electrical equipment mainly include lighting fixtures, air conditioning equipment, office equipment, and experimental instruments. The environment of different climate zones significantly impacts building carbon emission. However, when buildings are located in the same climate zone, the primary factor influencing their carbon emission is building functionality. The university has different building functions, energy-consuming equipment, and personnel density. Thus, university buildings have considerable differences in electricity consumption even if they were built at the same time. Moreover, their electricity consumption is closely related to the nature of their use. The statistical electricity consumption of buildings by category is shown in Fig. 3. The abscissa in the figure represents the number of buildings, the ordinate represents the power consumption intensity of buildings, and the bubble in the figure represents the power consumption. The larger the bubble is, the greater the power consumption of buildings.

The top three buildings in terms of energy consumption intensity are canteen, library, and engineering laboratories, with energy consumption intensities of 93.60, 78.35, and 44.39 kWh/m^2^, respectively. This finding is related to the large number of energy-consuming equipment, high energy consumption intensity, and long energy consumption time in these types of buildings. In addition, campus electricity consumption has evident temporal characteristics. Moreover, factors such as seasons, winter and summer vacations, and weekends significantly affect electricity consumption.

Transportation includes two parts: on-campus transportation and off-campus transportation, with a total emission of 7115.55 tCO_2_. On-campus transportation has evident tidal characteristics. Many teachers and students rush onto the campus roads during rush hour and large-scale events. From the perspective of transportation modes, motor vehicles, bicycles, and pedestrians interact with one another on some routes, resulting in a large number of mixed traffic between people and vehicles and reduced travel efficiency. Off-campus transportation includes student commuting, internships, and faculty travel. According to the student questionnaire survey, it was found that the main influencing factor for students to choose transportation is the distance of transportation. When the operating distance is less than 300 km, students mainly choose highways because of the following two factors. From a highway perspective, Henan Province is located in the center of China, with flat terrain, low bridge to tunnel ratios, and a well-developed highway network. Secondly, from the perspective of students, they can reach any county within the province without transferring from school. When the transportation distance is between 300 and 800 km, the longer the travel distance, the greater the possibility for students to choose railway transportation. This is because the safety, comfort, and punctuality of railways are higher than those of road transportation, and railway departments offer discounts for students to take trains. It is worth noting that the likelihood of students choosing regular trains is higher than that of high-speed railways, because students themselves are not yet financially independent and have a lot of freedom in terms of time, so regular trains are more favored by students. When the travel distance is greater than 800 km, although some air transportation methods have emerged, trains are still the main mode.

In terms of food, the consumption of plant- and animal-based foods by campus teachers and students is 9447.02 and 2208.92 kg, respectively. This finding indicates that vegetarian consumption plays an important role in daily dietary consumption on university campuses. However, the greenhouse gases released into the environment are generally high because of the high consumption of plants in the production process of animal-based foods. Moreover, the carbon emission intensity coefficient of these greenhouse gases is high. Although animal-based foods consume little, their carbon emission are large, accounting for 67.82% of food emissions. The carbon emission from paper includes student paper, teacher paper, and public paper, totaling 797.75 tCO_2_ emissions. In this value, the purchase of textbooks by students accounts for the largest proportion of approximately 70.51%, and the carbon emission of paper used for daily homework and teacher teaching are relatively low. The clothing emissions are 1803.13 tCO_2_, accounting for 2.67% of the total emissions on campus. The composition of campus waste is relatively simple, with food and kitchen waste being the main sources in the living area, waste paper and plastic bottles being the main sources in the teaching and office area, and packaging boxes being the main sources in the express delivery center. A visit to the school’s energy management office shows that nearly 20 tons of waste are produced per day and disposed of through a centralized landfill, resulting in an annual emission of 1701 tCO_2_.

### Comparison with other universities

The selection of calculation methods, content, and parameters may vary because of the different characteristics of each university. Thus, choosing a standard with which the carbon emission of different universities is compared becomes difficult. This article uses the maximum emission portions as the comparison standard. Table 2 summarized some campus carbon emission data from similar years. The concentration of accommodation and local climate may be the two main factors affecting carbon emission. Three universities have the highest commuting ratios, possibly because these universities do not provide on-campus accommodation. Moreover, students usually live off campus, thereby reducing energy consumption on campus but correspondingly increasing the carbon emission of teacher–student commuting. The local climate can also affect the carbon emission of universities to some extent. Given the high demand for heating, universities in cold regions often produce more carbon emission than those in warm regions. Finland and London are all located in cold regions. Thus, they have the highest proportion of thermal carbon emission. However, the American University of Sharjah is located in a tropical climate. Its largest source of carbon emission comes from electricity. This scenario may be related to the region’s need for a large amount of electricity for cooling.

**Table 2:** Carbon emission of some universities.

University name	Year	Population	Scope 1 %	Scope 2 %	Scope 3 %	Maximum emission source and its proportion	Total carbon emission (tCO_2_e)	Per capita carbon emission (tCO_2_e)	Ref
NED University of Engineering and Technology, Pakistan	2017	12,000	7.4	7.0	85.6	Commute, 84.2%	21,500	1.79	^35^
The American University of Sharjah, United Arab Emirates	2018/19	6041	0.37	61.12	38.51	Electricity, 60.91%	94,553.30	15.65	^36^
University of Oulu, Finland	2019	16,900	1.68	40.82	57.5	Heat,40.62%	19,072	1.13	^37^
North China University of Water Resources and Electric Power	2019	23,112				Heat, 51.29%	32,119.92	1.39	^38^
Universidad Nacional de Colombia, Medellín	2019	16,860	2.84	14.03	83.13	Transportation 58.51%	7,250.52	0.43	^39^
Bournemouth University, UK	2019	17,893	9	27	64	Commute, 54%	2,140	1.41	^40^
University of Ibadan, Nigeria	Jan.–Jun. 2019		4	90	6	Electricity, 90%	5,270.95	0.11	^41^
HPU	2019	43,137	6.46	61.27	32.27	Heat, 41.13%	65,327.44	1.51	This case
Chemical Engineering Department, Imperial College London, UK	2019/20		38	8	54	Combined heat and power, 41%	8,330		^42^
University of Bologna, Italian	2020	91,000	43	36	21	Natural gas	16,467	0.18	^43^

### Comparison with the national and city level

Guan^44^ calculated the Scope 1 carbon emission of China and its 30 provinces. Research shows that in 2019, the total carbon emission of China’s Scope 1 was 9.80 GtCO_2_, with a per capita of approximately 6.95 tCO_2_/person. When the calculation boundary is a country, the carbon emission of Scope 1 is close to the national carbon emission. The reason is that the vast majority of carbon emission are generated from fossil fuel combustion on-site and industrial production within its field, and imported electricity and heat are minimal. From the perspective of cities, the per capita emissions of 40 cities were calculated, with values ranging from 3.2 to 58.4^45^. Moreover, the emissions of Scope 1 and Scope 2 are close to half of the total emissions in terms of proportion, whereas the proportions of transportation and food in Scope 3 are relatively high. The calculation of campus carbon emissions should not be compared with the national or city level, but should be compared with regions with similar functions. Given the key differences between emission sources and assessment criteria, appropriate baseline targets include residential communities, commercial areas, public institutions or other campuses. This would lead to a more accurate and meaningful assessment, helping identify genuine emission issues and targeted reduction strategies.

### Carbon emission transfer

In addition to being classified according to the scope specified in the agreement, carbon emission can be divided by field. The purpose of field analysis is to identify the key carbon-emitting departments on campus and the departments that contribute the most to emission reduction, which are often key to low-carbon development. The main sources of campus carbon emission are energy, construction, transportation, food, paper, garbage, and other fields. The largest difference between the energy consumption structures of transportation and buildings is the proportion of electricity consumption. Electricity is the most important form of energy used in buildings, and the low-carbon development of the power system can effectively reduce carbon emission in the building field. In the transportation sector, oil products are still the main energy source. Therefore, the contribution of the power system’s low-carbon development to reducing carbon emission in the transportation sector is very limited if the proportion of new energy vehicles, such as electric vehicles, is not significantly increased. At present, electric vehicles use building electricity for charging. Thus, the carbon emission generated are calculated in the building field rather than the transportation field. This carbon emission transfer increases the proportion of building carbon emission. According to survey statistics and calculations, the amount of carbon emission transferred from the transportation field to the building field on the campus in 2019 was 1629.82 tons of CO_2_, accounting for 5.02% of the total transportation sector. In addition, global sustainable transportation has also grown with the rapid development of environmental protection technology and energy efficiency. At present, the fastest-growing industry is alternative driving technology. The sales of new energy electric vehicles will continue to increase in the future, and their proportion will gradually increase. Therefore, the carbon emission of the transportation field shifting to the building field will become increasingly significant.

### Emission reduction measures

#### Long term reduction measures

Regarding long-term measures, some higher education institutions have published carbon neutrality action plans (detailed in Table 1), primarily focusing on energy supply-side innovation, though most remain in the planning phase. Core technical pathways include: establishing an intelligent microgrid with wind-PV-storage coordination to achieve 100% renewable electricity supply; advancing building full-electrification retrofits to reduce end-use carbon emissions; deploying carbon capture facilities for laboratories and implementing full life-cycle carbon management systems. Current planned measures predominantly adopt multi-energy coupling technological approaches, as exemplified by Selcuk University^46^, University of Palermo^47^ and Cornell University^48^, demonstrating transitional characteristics from singular energy substitution toward systematic zero-carbon transformation.

**Fig. 4:**
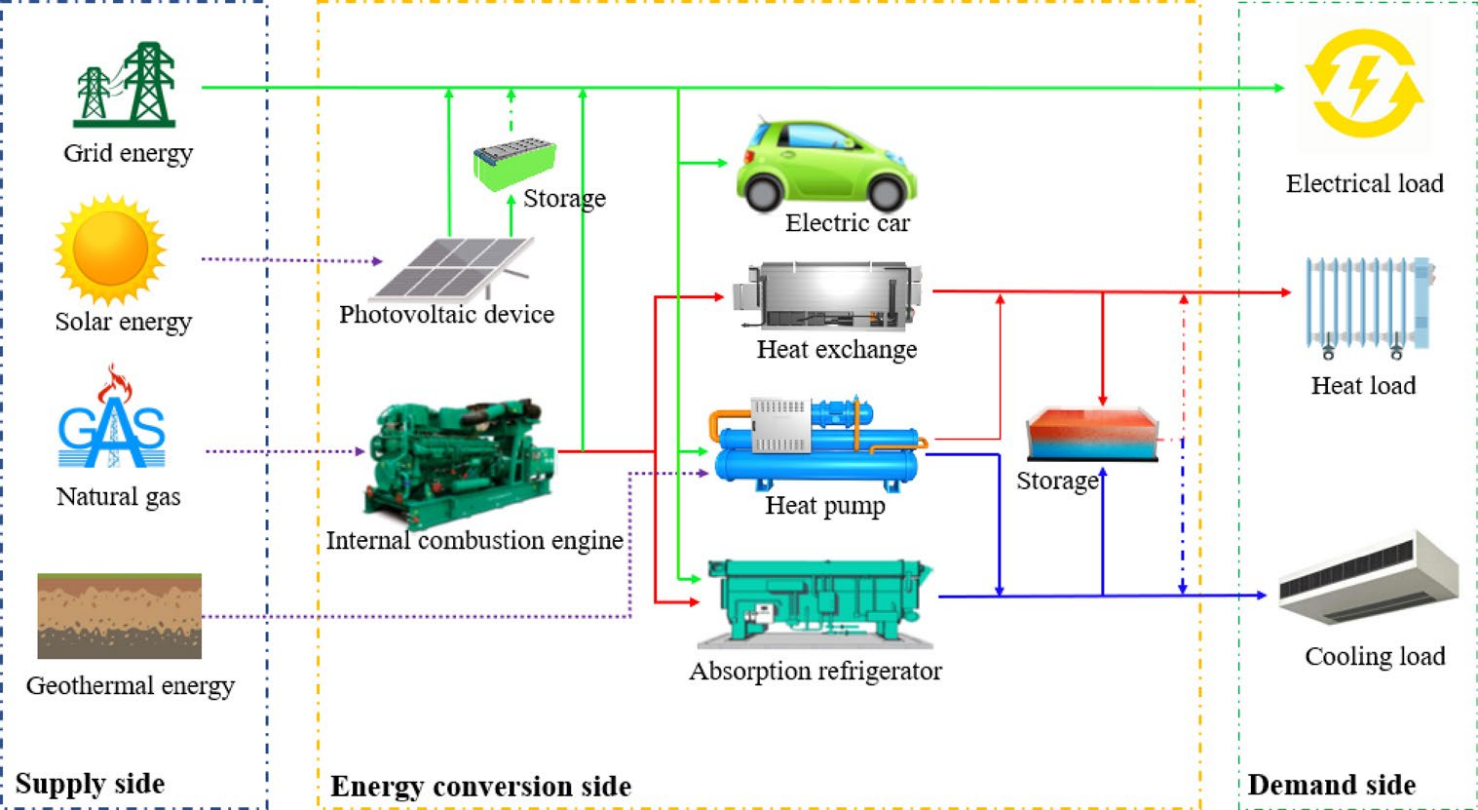
Conceptual diagram of the proposed multi-energy complementary system.

HPU occupies a large area with an open space and a small building plot ratio. In addition, the hydrogeological conditions of the campus include a thickness of more than 200 m in the quaternary system and a thickness of more than 30 m in the aquifer. Thus, it is a particularly suitable area for ground-source heat pumps. Jiaozuo City is hot in summer and cold in winter. This climate is conducive to achieving thermal balance and provides favorable conditions for utilizing shallow geothermal energy. In addition, the campus green area is relatively large, and the roof area is not fully utilized. Jiaozuo City has 2200–2400 h of sunshine throughout the year, with an annual total radiation of 4625.026–5020.026 MJ/m^2^. The abundant solar energy resources provide favorable conditions for the construction of photovoltaic power generation facilities. As shown in the Fig. 4, this study proposes a tailored energy coupling system scheme for the campus on the supply side on the basis of the actual situation of HPU. An important part of this is energy storage, where the unused electricity during the day can be used for lighting in public areas, streetlights, and charging electric vehicles at night.

With the objective of minimizing both the annual operating cost and carbon emission, a multi-objective optimization algorithm is used to optimize the configuration of various load scenarios. As a result, a multi-energy complementary system including 60,000 m^2^ solar panels, two internal combustion engines with a power of 5000 kW, three ground-source heat pumps with a power of 5000 kW, two waste heat boilers with a power of 4000 kW, and three lithium bromide absorption refrigeration units with a cooling power of 10,000 kW obtained. After the system is adopted, the annual carbon emission of Scope 1 + Scope 2 are 21876.32 tCO_2_, which is 49.44% of the original Scope 1 + Scope 2 annual carbon emission. The annual operating costs can be reduced by approximately $ 1.7 million. Moreover, vegetation can absorb CO_2_ in the atmosphere through photosynthesis during its growth process and retain it in vegetation or soil in the form of biomass. At present, the green area is approximately 32% of the total campus area and can absorb 10812.61 t of CO_2_. The emissions of Scope 3 depend on the types of emission sources it covers, with a great emphasis on individual consumer energy-saving behavior. Therefore, strengthening low-carbon and green training in daily teaching and life is necessary.

#### Short-term reduction measures

‌‌Short-term measures are currently focused on end-user energy efficiency enhancement, with technological pathways exhibiting diversified characteristics. Implementation approaches vary among universities; for example, Shenyang Jianzhu University adopted building envelope retrofits^49^, Universitat Politècnica de Catalunya utilized waste heat recovery technology^50^, and five Chinese universities implemented biomass energy applications^51^. Additionally, behavioral intervention mechanisms (green mobility initiatives, individual energy-saving incentives) and carbon management technologies (small-scale carbon capture units, carbon-sink landscape development) were collectively constituted as short-term decarbonization portfolios. Despite high technological maturity, implementation efficacy was significantly influenced by variables including regional climate and energy consumption patterns.

In the foreseeable future, the supply-side transformation may not be implemented quickly, and the demand-side transformation for energy conservation will remain the most important means of carbon reduction. The energy-saving renovation measures on the demand side of the campus aim to optimize the use and management of campus energy, reduce energy consumption, improve energy efficiency, and achieve sustainable development of the campus. In terms of heating, the insulation performance of buildings must be improved, and air tightness must be enhanced to reduce the heating load. For electricity, the metering and charging regulations should be strengthened, low-energy equipment should be used, and energy-saving renovations should be performed. In terms of water resources, we should save water, utilize reclaimed water, and build sponge campuses. In terms of transportation, planning routes reasonably, conducting video conferences, and promoting green travel are necessary. As far as food is concerned, we should save energy on food, put an end to waste, and carry out the Clean Your Plate Campaign. Regarding garbage, we must strengthen classification and recycling systems and study the utilization of renewable resources. For paper, advocating paperless teaching and office work and reusing old books are necessary. Moreover, actively and reasonably utilizing renewable energy, increasing green plant areas, and promoting a low-carbon campus culture are recommended.

Long-term planning for carbon-neutral campuses must maintain strategic rigidity, while short-term implementation requires tactical flexibility. The two are dynamically calibrated to achieve the synergistic dual goals of “emission reduction-education” unique to campuses.

### Limitations and future research

The uncertainty in carbon emission calculation comes from the uncertainty of activity data and emission factors. Many factors affect the results of campus carbon emission accounting, such as emission factors and activity levels, omissions, or duplicate calculations. These factors can all affect the accuracy and reliability of the accounting results. In this study, some energy activities such as business trips and commuting mileage were not collected completely because of the use of data collection methods. As a result, the calculation data are relatively small. From the perspective of the whole life cycle, this study did not consider the carbon emission of building material production and construction. This scenario also leads to a small value of the carbon emission. Additionally, this study presented multi-objective optimization results, yet the designed multi-energy complementary system has not been implemented.

Moreover, in recent years, artificial intelligence (AI) technology was widely used in various fields, including carbon emission accounting. Research showed that AI technology could significantly reduce carbon emission levels. AI technology had two main applications. One was to utilize various optimization algorithms, such as the long short-term memory model^52^, ResNet BIGRU-TPA network^53^, and Visual Question Answering Models^54^, which employed historical data to estimate future carbon emission trends. Additionally, through AI algorithm integration of Internet of Things equipment data, precise accounting and dynamic visualization of campus carbon emission were achieved. The second approach involved constructing prediction models based on the characteristics of campus scenarios, where building information modeling and remote sensing image processing technologies were applied to simulate emission reduction pathways via digital twin technology^55,56^. ‌In further research, the energy consumption trend could be predicted and the emission reduction path optimized through machine learning, with the carbon emission reduction effect in different scenarios simulated through the integration of digital twin technology.

## Conclusion

Through a case study of HPU South Campus, the main sources of carbon emissions were identified, the relationships between different campus sections were studied, and targeted emission reduction measures were proposed. In 2019, the carbon emission of the campus was mainly caused by energy use, with thermal, electricity, and natural gas sources accounting for 42.46%, 18.81%, and 6.34%, respectively. Research has shown that a transfer of carbon emission exists between different fields, particularly from the transportation field to the building field. Moreover, 81.52 tons of carbon emission have been transferred to this campus, and the amount of carbon emission transfer will be even greater in the future.

As microcosmic societal units that carry both demonstrative significance and practical value in low-carbon transition under carbon neutrality goals, universities must develop context-specific long-term and short-term emission reduction strategies tailored to actual campus conditions. On the energy supply side, this study proposes a multi-energy complementary system, which reduces carbon emission by 49.63% annually and saves approximately $ 1.7 million in operating costs. In addition, the absorption of green plants greatly reduces carbon emission.

Universities should implement AI-driven systems to establish precise carbon quantification frameworks, predict campus energy consumption patterns, and formulate dynamic emission reduction strategies via digital twin-enabled simulation platforms. This study supports universities in implementing carbon neutrality policies by assessing current emissions and advancing zero-carbon campuses.

## Supplementary Information

Below is the link to the electronic supplementary material.

Supplementary Material 1

## Data Availability

All data generated or analyzed during this study are included in this published article and its supplementary information files.
