# Supplementary material for: Accounting carbon emission and proposals for their reduction at a university campus in China
Source: Sci Rep. 2026 Mar 23;16:14546. doi: 10.1038/s41598-025-23719-z (PMC13153179; doi:10.1038/s41598-025-23719-z)
Supplement: Supplementary file 1 — Supplementary Material 1 [file 41598_2025_23719_MOESM1_ESM.docx]

Supplementary Material

All carbon emission activity data and its factor shown in Table.S1.

Table.S1 Carbon emission data inventory of HPU and carbon emission factors

| Sector | Emission source | Consumption(unit) | GHG emission factor(unit) |
| --- | --- | --- | --- |
| Energy | Heating | 252,178 GJ | 0.11 tCO_2_/GJ [S1] |
|  | Natural gas | 1,895,802 m^3^ | 21.84 tCO_2_/10^4^Nm^3^ [S1] |
|  | Electricity | 23,371M⋅Wh | 0.5257 tCO_2_/M⋅Wh [S1] |
|  | Water | 325,110 km^3^ | 0.168 kg CO_2e_/ton [S2] |
| Transportation | Light-duty trucks | 556,680 kg⋅km | 0.14 kg CO_2e_/ton⋅km [S3] |
|  | Medium-duty trucks | 2,268,892 kg⋅km | 0.065 kg CO_2e_/ton⋅km [S3] |
|  | Expressway | 67,495,200 km | 0.075 kg CO_2e_/km [S4] |
|  | Ordinary-speed rail | 39,573,000 km | 0.012 kg CO_2e_/ km [S4] |
|  | High-speed rail | 28,320,600 km | 0.021 kg CO_2e_/km [S4] |
|  | Air travel (medium haul) | 1,110,714 km | 0.097 kg CO_2e_/km [S4] |
|  | Air travel (long haul） | 7,478,762 km | 0.087 kg CO_2e/_km [S5] |
| Food | Wheat | 3,126,142 kg | 0.27 kg CO_2e_/kg [S5] |
|  | Rice | 756,297 kg | 0.27 kg CO_2e_/kg [S6] |
|  | Edible oil | 395,190 kg | 1.48 kg CO_2e_/kg [S6] |
|  | Vegetables | 2,915,604 kg | 0.4 kg CO_2e_/kg [S6] |
|  | Pork | 405,113 kg | 7.64 kg CO_2e_/kg [S6] |
|  | Beef | 95,778 kg | 12.04 kg CO_2e_/kg [S6] |
|  | Mutton | 38,829 kg | 18.86 kg CO_2e_/kg [S6] |
|  | Poultry | 191,124 kg | 1.71 kg CO_2e_/kg [S6] |
|  | Aquatic products | 89,306 kg | 1.94 kg CO_2e_/kg [S6] |
|  | Eggs | 613,062 kg | 0.78 kg CO_2e_/kg [S6] |
|  | Dairy | 775,711 kg | 0.36 kg CO_2e_/kg[S6] |
|  | Dried and fresh fruits | 2,016,935 kg | 0.07 kg CO_2e_/kg[S6] |
|  | Sugar | 236,855 kg | 0.08 kg CO_2e_/kg [S6] |
| Clothes | | 43,137 | 41.8 kg CO_2e_/person [S7] |
| Paper | | 570 t | 1.4 tCO_2e_/ton [S8] |
| Waste | | 6,300 t | 0.27 tCO_2e_/ton[S9] |

S1. Administration for Market Regulation of Henan Province, China, General Rules for Reporting Carbon Dioxide Emissions Information (DB41/T 1710-2018), (DB41/T 1710-2018), China Planning Press, Beijing, 2018

S2. Ministry of Housing and Urban-Rural Development of People’s Republic of China, Carbon emission calculation standard for buildings (GB/T51366-2019), China Planning Press, Beijing, 2019

S3. X.Z. Zhang, X. Hao, Y. Liu, R. Wu, X.N. Shan, S.X. Li, Contribution of potential clean trucks in carbon peak pathway of road freight based on scenario analysis: A case study of China, J Clean Prod. 379134669 (2022).

S4. P.H. Chen, Y. Lu, Y.L. Wan, A.M. Zhang, Assessing carbon dioxide emissions of high-speed rail: The case of Beijing-Shanghai corridor, Transp Res D Transp Environ. 97102949 (2021).

S5. L. Vasquez, A. Iriarte, M. Almeida, P. Villalobos, Evaluation of greenhouse gas emissions and proposals for their reduction at a university campus in Chile, J Clean Prod. 108(DEC.1PT.A),924-930 (2015).

S6. C. Zhihong, H. Jinmin, X. Hongping, Spatial-temporal change of Chinese resident food consumption carbon emissions and its driving mechanism (in Chinese), Progress in Geography. 39(1),91-99 (2020).

S7. Quantis, Environmental Impact of the Global Apparel and Footwear Industries Study, 2018. https://refashion.fr/eco-design/sites/default/files/fichiers/Measuring%20Fashion%20Environmental%20Impact%20of%20the%20Global%20Apparel%20and%20Footwear%20Industries%20Study.pdf.

S8. Y.T. Wang, X.C. Yang, M.X. Sun, L. Ma, X. Li, L. Shi, Estimating carbon emissions from the pulp and paper industry: A case study, Appl Energy. 184779-789 (2016).

S9. The World Resources Institute, GHG Accounting Tool for Chinese Cities, 2015. https://wri.org.cn/research/greenhouse-gas-accounting-tool-chinese-citiespilot-version-10.

This study takes travel distance and mode as the factor to obtains the transportation data of college students.

The questionnaire contents

(1) The mode of transportation for you to return to school during vacation is

A: road B: ordinary-speed rail C: high-speed rail D: air travel

(2) How far is your commute distance in winter and summer vacation?

A: <300 km; B: 300‒800 km; C: >800 km

(3) The number of times you buy online in a year:

(4) Product dispatching point of purchased goods:

(5) Weight of purchased goods:

Questionnaire survey results

A total of 500 questionnaires were sent out in this survey, and the actual number of valid questionnaires collected was 442, accounting for 88.4%. The survey transportation modes result is shown in the Table.S2.

Table S2 Number of students surveyed with different transportation modes.

|  | High-speed road | Ordinary-speed rail | High-speed rail | Aviation | Total number |
| --- | --- | --- | --- | --- | --- |
| <300 km | 101 | 70 | 21 |  | 192 |
| 300-800 km | 50 | 77 | 26 | 4 | 157 |
| >800 km | 0 | 31 | 54 | 8 | 93 |

Based on the proportion and distance obtained from the survey results, the total campus transportation data were calculated. The result shown is Table S3.

Table S3 Traffic data.

| Mode of travel | Average mileage (km) | Number | Weight (t) | Frequency | Unit energy consumption (MJ/pkm, MJ/tkm) | Energy source | Consumption |
| --- | --- | --- | --- | --- | --- | --- | --- |
| High-speed train | 300 | 3,535 |  | 6 | 0.42 | Electricity (kW·h) | 742,350 |
|  | 800 | 812 |  | 6 | 0.42 | Electricity (kW·h) | 454,720 |
|  | 1,200 | 2,377 |  | 6 | 0.42 | Electricity (kW·h) | 1,996,680 |
| Faculty train | 1,200 | 1,600 |  | 2 | 0.42 | Electricity (kW·h) | 448,000 |
| Ordinary train | 300 | 11,615 |  | 6 | 0.28 | Diesel oil (t) | 137.08 |
|  | 800 | 2,433 |  | 6 | 0.28 | Diesel oil (t) | 76.57 |
|  | 1,200 | 1,376 |  | 6 | 0.28 | Diesel oil (t) | 64.96 |
| Bus | 300 | 16,540 |  | 8 | 1.4 | Diesel oil (t) | 1,301.36 |
|  | 800 | 1,587 |  | 6 | 1.4 | Diesel oil (t) | 249.73 |
| Chartered bus for practice | 120 | 24,000 |  | 1 | 1.4 | Diesel oil (t) | 94.42 |
| Private car | 6,000 | 1,847 |  |  | 2.45 | Petrol (t) | 629.60 |
| Aviation | 6,800 | 398 |  | 2 | 4.90 | Aviation kerosene (t) | 594.81 |
| canteen truck | 5 |  | 5 | 48*2 | 0.58 | Diesel oil (t) | 0.06 |
| Supermarket truck | 30 |  | 30 | 48*3 | 0.58 | Diesel oil (t) | 3.03 |
| Garbage truck | 35 |  | 18 | 50*7 | 0.58 | Diesel oil (t) | 5.16 |
| Express truck | 600 | 43,143 | 0.003 | 27.3 | 0.58 | Diesel oil (t) | 50.79 |

The results of the frequency, distance, and item information of online shopping are shown in Tables S4, S5, and S6, respectively.

Table S4 Statistical results of online shopping times.

| Frequency | <5 | 6~10 | 11~15 | 16~20 | 21~26 | 26~30 | 31~35 | 36~40 | 41~45 | >45 | Total |
| --- | --- | --- | --- | --- | --- | --- | --- | --- | --- | --- | --- |
| Number | 26 | 42 | 42 | 65 | 40 | 38 | 50 | 46 | 33 | 60 | 442 |
| Proportion (%) | 5.88 | 9.50 | 9.50 | 14.71 | 9.05 | 8.60 | 11.31 | 10.41 | 7.47 | 13.57 | 100 |

Table S5 Distribution of transportation distance for online shopping.

|  | 0~300 | 300~1000 | 1000~2000 | >2000 |
| --- | --- | --- | --- | --- |
| Proportion (%) | 23.08% | 29.19% | 35.52% | 12.22% |

Table S6 Online purchase of goods.

| Product Type | Proportion (%) |
| --- | --- |
| Clothing accessories | 28.23 % |
| Electronic products | 16.84 % |
| Cultural and sports goods | 23.47 % |
| Snacks | 8.69 % |
| Daily necessities | 13.77 % |
| Virtual products | 1.82 % |
| Others | 7.18 % |
| Ttotal | 100 % |

The questionnaire survey revealed the average annual shopping frequency of students to be 27.3 times. The shopping items are mainly clothing accessories, electronic products, cultural and sports articles, and snacks, which are small to medium in size. The online consumption structure of students is gradually showing a diversified trend, with a variety of types, but there are no large items such as furniture and household appliances. The average weight is calculated at 3 kg/piece in the paper.
